# Refer-ASV: Referring Multi-Object Tracking in Autonomous Surface Vehicle Navigation Scenes

**DOI:** 10.3390/jimaging12040145

**Published:** 2026-03-25

**Authors:** Bin Xue, Qiang Yu, Kun Ding, Ying Wang, Shiming Xiang, Chunhong Pan

**Affiliations:** 1 State Key Laboratory of Multimodal Artificial Intelligence Systems, Institute of Automation, Chinese Academy of Sciences, Beijing 100190, China; xuebin2018@ia.ac.cn (B.X.); qiang.yu@ia.ac.cn (Q.Y.); ying.wang@ia.ac.cn (Y.W.); smxiang@nlpr.ia.ac.cn (S.X.); chpan@nlpr.ia.ac.cn (C.P.); 2 School of Artificial Intelligence, University of Chinese Academy of Sciences, Beijing 101408, China

**Keywords:** referring multi-object tracking, water-surface scenes, autonomous surface vehicles, dataset construction

## Abstract

Water-surface perception is critical for autonomous surface vehicle navigation, where reliable tracking of task-relevant objects is essential for safe and robust operation. Referring multi-object tracking (RMOT) provides a flexible tracking paradigm by allowing users to specify objects of interest through natural language. However, existing RMOT benchmarks are mainly designed for ground or satellite scenes and fail to capture the distinctive visual and semantic characteristics of water-surface environments, including strong reflections, severe illumination variations, weak motion constraints, and a high proportion of small objects. To address this gap, we introduce Refer-ASV, the first RMOT dataset tailored for ASV navigation in complex water-surface scenes. Refer-ASV is constructed from real-world ASV videos and features diverse navigation scenes and fine-grained vessel categories. To facilitate systematic evaluation on Refer-ASV, we further propose RAMOT, an end-to-end baseline framework that enhances visual–language alignment throughout the tracking pipeline by improving visual–language alignment and robustness in challenging maritime environments. Experimental results show that RAMOT achieves a HOTA score of 39.97 on Refer-ASV, outperforming existing methods. Additional experiments on Refer-KITTI demonstrate its generalization ability across different scenes.

## 1. Introduction

Water bodies cover approximately 70% of the Earth’s surface, and a wide range of modern activities, including international trade, resource exploration, and military operations, take place in water scenes [1]. With the rapid progress of deep learning, intelligent perception over water surfaces has advanced significantly, enabling the deployment of autonomous surface vehicles (ASVs) in practical applications such as floating waste cleaning [2], maritime search and rescue [3], and recreational operations [4]. Related studies on UAV-based garbage detection [5], UAV-network localization [6], and real-time vehicle-data-driven traffic prediction [7] further highlight the growing importance of intelligent perception in unmanned and transportation systems. These advances emphasize the need for reliable perception of surrounding objects in complex dynamic scenes. In ASV-based water-surface perception tasks, such as object detection [8] and multi-object tracking (MOT), tracking plays a critical role by enabling continuous monitoring of surrounding objects and supporting key functions such as navigation and path planning. However, most existing water-surface MOT studies [9] focus on tracking all observable objects in a scene. In real autonomous navigation scenes, not all water-surface objects are equally relevant at every moment. Instead, users often need to focus on objects with specific motion patterns, spatial states, or semantic attributes. For example, during autonomous navigation, anchored vessels that may obstruct planned routes require close monitoring, while during harbor entry and berthing, vessels exhibiting particular headings or maneuvering behaviors become primary objects of interest. These practical requirements call for more flexible and interactive tracking paradigms, in which tracking objectives can be specified according to user intent. Motivated by this need, referring multi-object tracking (RMOT) has been introduced for ASV navigation scenes. The RMOT task aims to continuously localize and track all water-surface objects that match a given natural language description [10].

As an emerging and promising task, RMOT has developed rapidly in recent years. However, existing RMOT benchmarks are mainly designed for road or ground scenes, where visual layouts are relatively structured and object motions are strongly constrained by traffic rules and fixed infrastructure. In contrast, water-surface scenes encountered by ASVs exhibit challenges that differ substantially from those of ground scenes. From an imaging perspective, water-surface scenes are characterized by complex shoreline backgrounds, frequent water reflections, pronounced illumination variations, and adverse conditions such as water mist [11]. Moreover, the large spatial extent of water surfaces results in a high proportion of small and distant objects. From the perspective of object properties and behaviors, water-surface objects are subject to weaker motion constraints and show less predictable dynamics. Objects may drift in multiple directions or remain anchored, while being continuously influenced by environmental factors such as water currents, reflections, and nearby structures. As a result, referring expressions in water-surface RMOT must capture water-specific behaviors, environmental conditions, and object–environment interactions. These characteristics are largely absent from existing RMOT benchmarks, and this lack of data has become a major obstacle to the development of water-surface RMOT.

The purpose of this study is to investigate RMOT in realistic water-surface navigation scenes from both the benchmark and method perspectives. To address the above limitations, we construct Refer-ASV, an RMOT dataset designed for ASV navigation in complex water-surface scenes. Unlike existing RMOT datasets, Refer-ASV explicitly focuses on visual and semantic characteristics that are specific to water-surface scenes. Specifically, Refer-ASV has the following properties: (1) complex water-surface conditions, including reflections, wave-induced motion, water spray, cluttered backgrounds, and a high proportion of small objects; (2) diverse real-world ASV navigation scenes, covering both inland waters and open-sea environments, with multiple vessel categories and water-surface objects, all collected from ASV platforms; and (3) application-oriented referring expressions aligned with practical water-surface tasks, featuring fine-grained vessel categories, explicit environmental descriptions, and water-specific object behaviors, such as drifting, anchoring, and object–environment interactions. Refer-ASV consists of 29 video sequences with 10,747 frames and 2,358 referring expressions, enabling systematic evaluation of RMOT methods under realistic water-surface conditions.

Moreover, to facilitate systematic evaluation of the proposed benchmark, we introduce RAMOT, an end-to-end baseline method tailored for water-surface RMOT. Due to the characteristics of water-surface scenes, effective RMOT in this setting requires stable visual–language associations throughout the tracking pipeline. However, such associations are often undermined by sparse supervision during training, semantic inconsistency across multi-scale visual representations, and unreliable tracker predictions under degraded imaging conditions. Based on these observations, RAMOT enhances visual–language alignment at three stages, namely, training, feature representation, and inference. Specifically, inspired by curriculum learning [12], we introduce an expression sampling strategy that stabilizes early cross-modal learning by progressively transitioning from simple expressions to more complex semantics. To handle the large scale variations of water-surface objects, we further propose a scale-aware fusion mechanism that preserves semantic consistency across different visual resolutions. In addition, an adaptive query management strategy is designed to recover high-confidence tracklets and improve robustness under degraded language cues or challenging imaging conditions.

Therefore, the objective of this study is twofold: to establish a dedicated referring multi-object tracking benchmark for autonomous surface vehicle navigation scenes and to develop a strong end-to-end baseline for systematic evaluation on this benchmark. Accordingly, this work focuses on two core questions: how to build a realistic dataset that captures the visual and semantic characteristics of water-surface scenes, and how to improve visual–language alignment for robust tracking under sparse supervision, scale variation, and degraded imaging conditions. In terms of methodology, we combine benchmark construction based on real-world shipborne videos with a Transformer-based end-to-end tracking framework and validate the proposed solution through comprehensive experiments on both water-surface and road-scene benchmarks. Experimental results show that the proposed framework achieves strong performance on Refer-ASV and generalizes effectively across different scenes. Our contributions are summarized as follows:

1. We are the first to systematically introduce RMOT into complex water-surface scenes encountered in ASV navigation.
2. We construct Refer-ASV, the first dataset dedicated to RMOT in complex water-surface scenes. It features fine-grained vessel categories, explicit environmental descriptions, and water-specific object behaviors.
3. We analyze the characteristics of Refer-ASV and investigate the challenges posed by water-surface scenes for RMOT through extensive experimental evaluation.
4. We propose RAMOT, a baseline model specifically designed for water-surface RMOT and demonstrate its effectiveness on the Refer-ASV dataset.

The remainder of this paper is organized as follows. Section 2 reviews related work on referring multi-object tracking, water-surface perception datasets, and degraded-scene detection methods. Section 3.1 introduces the Refer-ASV dataset and its construction process. Section 3.2 presents the proposed RAMOT framework and its key components. Section 4 reports the experimental setup and evaluation results. Finally, Section 5 concludes the paper and discusses future research directions.

## 2. Related Work

Our work focuses on water-surface RMOT, which lies at the intersection of three closely related research directions, namely, MOT, RMOT, and text-driven perception in water-surface scenes. These directions are complementary in our problem setting: MOT provides the fundamental framework for temporal object localization and association, RMOT introduces language-guided tracking paradigms and benchmarks, and text-driven perception in water-surface scenes reflects the growing need for language-conditioned perception in maritime environments. To situate our study within this research context, this section reviews representative work on MOT, RMOT methods and benchmarks, and recent advances in text-driven perception tasks related to water-surface scenes.

### 2.1. Multi-Object Tracking

Multi-object tracking (MOT) is a classical vision problem that has been extensively studied. Its goal is to localize all objects of interest in video sequences while maintaining consistent identities over time [13]. Existing MOT methods can generally be divided into two main paradigms according to how detection and tracking are organized: track-by-detection (TBD) methods [14,15,16] and end-to-end methods [17,18,19,20]. TBD methods decouple object detection and tracking, first detecting objects in each frame and then performing data association as a separate step. Representative studies improve tracking performance mainly through more robust appearance modeling and data association strategies [14,15,16]. In contrast, end-to-end methods jointly optimize detection and identity association within a unified framework, enabling shared feature learning across subtasks [17,18,19,20]. Recently, Transformer-based approaches have received increasing attention because of their strong capability for sequence modeling. Representative works include TransCenter [21], MOTR [22], and STDFormer [23], which leverage Transformer architectures to model object queries and temporal associations. Among them, MOTR [22] has become an important foundation for many subsequent end-to-end referring multi-object tracking frameworks.

While most MOT studies focus on road or ground scenes, several works have also explored water-surface scenes. From a methodological perspective, Sea-IoUTracker [9] introduces motion modeling and constraint mechanisms tailored to maritime scenes to address severe appearance variations and tracking instability in sea-surface scenes. VTM-YS [24] incorporates temporal association modeling and feature enhancement strategies to mitigate frequent object loss and unstable associations of small-scale vessels under complex sea conditions. From a data perspective, SMD [25] mainly targets ocean scenes collected from shore-based platforms. PoLaRIS [26] and USVTrack [27,28] are built on shipborne data. PoLaRIS includes multiple sensing modalities, such as infrared, and focuses on object tracking near ports. In [27], the authors propose a system that integrates RGB and millimeter-wave radar data and is designed for inland waterway tracking. The authors of [28] cover diverse water-surface scenes, including inland waters and marine scenes. Despite the progress achieved by water-surface MOT research, these methods aim to track all objects in a scene and cannot selectively track objects with specific semantic attributes according to user intent.

### 2.2. Referring Multi-Object Tracking

Referring multi-object tracking (RMOT) integrates natural language instructions with video-based tracking [10]. Its goal is to continuously localize all referring objects in a scene, shifting tracking from category-agnostic detection to language-guided object perception. Existing RMOT methods mainly address visual–language mismatch through multimodal feature fusion at different stages and can be broadly divided into two pipelines: end-to-end frameworks and two-stage track-then-refer pipelines. End-to-end methods, such as TransRMOT [10], TempRMOT [29], MGLT [30], and CDRMT [31], are built upon MOTR [22] and enhance text-guided tracking by improving multimodal fusion and temporal modeling within a unified architecture. In contrast, two-stage methods such as iKUN [32] decouple tracking and language grounding, resulting in a more modular and flexible design.

In addition, several RMOT benchmarks have been introduced in recent years. Refer-KITTI [10] and Refer-KITTI-V2 [29] extend the KITTI dataset [33] with language descriptions and focus on road traffic and autonomous driving scenes. Refer-Dance [32], built upon DanceTrack [34], targets human-centric activity scenes such as dancing. GroOT [35] further expands RMOT to a broader range of terrestrial natural environments. Beyond ground-based benchmarks, RMOT-SV [36] has recently been proposed for remote sensing scenes, providing a top-down observation perspective. Despite these advances, existing RMOT benchmarks primarily target ground-based or aerial scenes, and RMOT in shipborne water-surface scenes relevant to ASV navigation remains underexplored, even though it is critical for ASV autonomy and human–machine interaction.

### 2.3. Text-Driven Perception in Water-Surface Scenes

The growing importance of human–machine interaction in water-surface scenes has motivated recent studies on text-driven perception in such scenes. WaterVG [37] introduces textual input into water-surface perception by combining natural language prompts with multi-sensor data, including cameras and 4D millimeter-wave radar, to perform visual grounding in inland waterway scenes through object detection and mask prediction. Building on this direction, NanoMVG [38] focuses on referring visual grounding on water surfaces under resource constraints, unifying camera, radar, and language information via lightweight multimodal fusion. As a complementary line of work, WaterCaption [39] and the associated Da Yu model extend text-centric perception to scene-level captioning in water environments, producing descriptive narratives of waterway scenes. Although these studies advance text-driven perception in water-surface settings, they are limited to image-level grounding or captioning and do not model temporal dynamics in video, leaving a gap with the continuous scenes encountered in real-world ASV operations.

## 3. Data and Methods

This section presents both the constructed Refer-ASV dataset and the proposed RAMOT framework. We first describe the dataset construction process and its statistical characteristics, and then introduce the overall method design and the key modules developed for water-surface RMOT.

### 3.1. Refer-ASV Dataset

This subsection describes the construction process of the Refer-ASV dataset, including data collection, expression annotation strategies, and dataset splitting protocols. We further conduct a statistical analysis of Refer-ASV to provide an intuitive overview of its characteristics.

#### 3.1.1. Data Collection

Due to the limited availability of acquisition platforms, restricted geographic coverage, high collection costs, and safety risks, shipborne water-surface data are far less abundant than road traffic data. To reduce data collection costs while maintaining scene diversity, we constructed the water-surface RMOT dataset based on existing open-source ASV MOT benchmarks [27,28]. During data selection, we emphasized diversity in real-world ASV navigation scenes, covering multiple water regions and including scenes with complex backgrounds, dense objects, diverse object behaviors, and degraded imaging conditions. Following these criteria, we adopted [28] as the primary data source. This dataset was collected by shipborne cameras across a wide range of inland and maritime scenes and features high object density, with small objects accounting for over 30%, complex motion patterns, and diverse degradation factors such as water spray, surface reflections, and mirror effects. However, scenes dominated by water mist are relatively underrepresented. In contrast, the dataset Ref. [27] was mainly collected in inland river environments and contains a large number of degraded scenes with water mist and low illumination but exhibits more homogeneous scenes and lower object density. To complement these conditions, we selected sequences 21 and 24 from [27]. In total, the collected data comprised 29 sequences with 10,747 frames. Tracking annotations followed the MOT Challenge format [40], where each frame included object category labels, bounding boxes, and object identities.

Overall, the current version of Refer-ASV mainly covers daytime normal and degraded water-surface scenes, including water-mist, cloudy, low-illumination, and overexposed conditions. Dedicated nighttime or extremely dark environments are not yet included, mainly due to the scarcity of shipborne water-surface data and the difficulty of data collection in real-world ASV navigation scenes.

#### 3.1.2. Data Annotation and Dataset Split

Inspired by [29], we adopted an efficient annotation pipeline to enrich the collected MOT data with high-quality referring expressions while incorporating semantics specific to water-surface scenes. The pipeline was designed to progressively increase the semantic richness of expressions and consisted of three stages: annotating basic semantic phrases that describe water-surface object attributes, generating complete expressions based on predefined grammatical rules, and refining and expanding the generated expressions using a large language model (LLM).

**(1) Annotation of basic semantic phrases for water-surface object attributes.** We first annotated descriptive terms that characterized object attributes. Unlike existing RMOT benchmarks, whose expressions typically focus on appearance, action, and location using generic natural-scene vocabulary, our annotations incorporated semantics specific to water-surface environments and more complex interactions. These included environmental descriptions, fine-grained vessel categories, and interactions with other objects or the surrounding environment. Specifically, the basic semantic phrases in Refer-ASV are organized into five categories. *Appearance* includes fine-grained vessel types (e.g., duck boats and yachts), color attributes (both primary and mixed colors), and relative size with respect to other objects. *Attributes* describe interactions with other objects and water-specific characteristics (e.g., people standing onboard and clear water reflections). *Actions* cover both general motions and water-specific behaviors, such as moving toward each other, entering a harbor, anchoring, and passing under a bridge. *Environment* includes weather and illumination conditions, such as water spray and strong sunlight. *Location* covers general positions as well as water-specific spatial descriptions, such as left or right, near the left bank, and in the middle of the water. During annotation, we developed an interactive labeling tool inspired by the annotation pipeline in [29], which was independently implemented for our dataset. Given a video sequence with tracking box annotations, the tool displays all tracked boxes frame by frame. Annotators input a basic semantic phrase and select the start and end boxes of objects that match the description. The tool then automatically records all corresponding tracking annotations within the specified frame interval. After processing a sequence, the tracking annotation file associated with the basic phrase is saved.

**(2) Grammar-based expression generation.** After obtaining the basic semantic phrases and their corresponding annotations, we generated complete referring expressions using 12 predefined templates based on grammatical rules. These templates were derived from five basic template patterns through different combinations. Table 1 summarizes the basic templates, which can be flexibly combined to produce diverse expression variants, ranging from concise descriptions to longer expressions composed of multiple semantic elements. For each generated expression, we defined its tracking label as the set of object tracklets that satisfy the expression over time. Specifically, the tracking label of an expression was constructed as the intersection of the tracklet sets associated with its constituent basic semantic phrases:

(1) $$Y\left(e\right)=\overset{K_{e}}{\underset{k=1}{⋂}}Y\left(p_{k}\right),$$

where *e* denotes a referring expression, $p_{k}$ denotes the *k*th basic semantic phrase composing *e*, Y(·) returns the set of object tracklets annotated for a given phrase or expression, and $K_{e}$ is the number of basic phrases in the expression. In addition, during expression generation, we retained a small proportion (5%) of expressions that did not refer to any object to increase the presence of negative samples. Using the predefined templates, a total of 922 basic expressions were generated.

**(3) LLM-based expression refinement and expansion.** Expressions generated by fixed grammatical rules tend to be rigid and differ from natural human language. To correct grammatical issues and improve linguistic fluency while preserving the original semantics, we employed Qwen-3 [41] to expand each basic expression into three variants with different sentence structures but consistent meanings. This refinement step increased lexical and syntactic diversity without altering the underlying object semantics or tracking labels.

**Figure 1:**
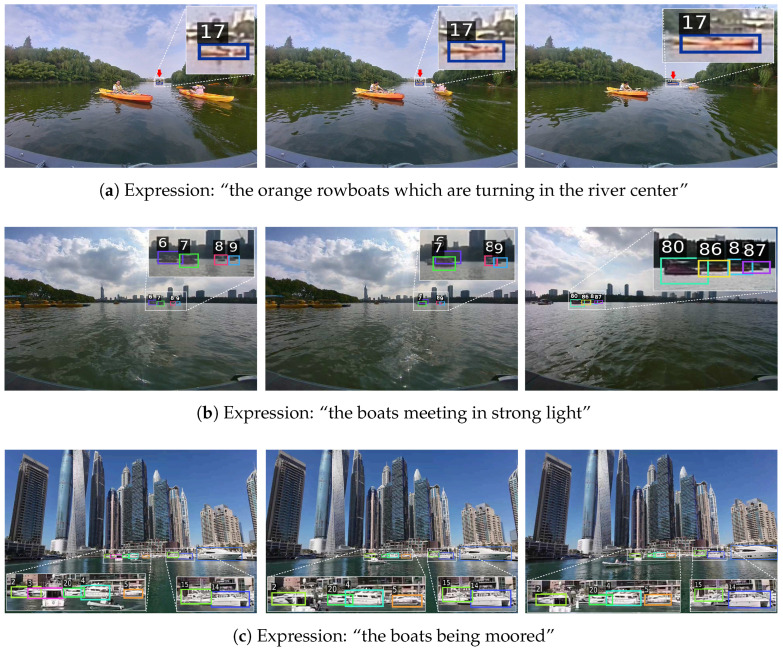
Representative examples from Refer-ASV. Boxes in different colors indicate different referring objects. Best viewed when zoomed in.

After the semi-automatic annotation pipeline, multiple rounds of manual verification were performed by domain experts to ensure expression fluency and accurate alignment with object semantics. Specifically, the initial annotation and the final full-dataset check were completed by the first author using a unified annotation standard. Three domain experts from related research areas then conducted cross-checking on different subsets of the dataset, and the reviewed subsets were subsequently exchanged for additional verification. As a result, Refer-ASV contains 2358 referring expressions. Following the dataset splitting protocol in [27,28], the training set includes 15 sequences with 6761 frames, while the test set consists of 14 sequences with 3986 frames, corresponding to 1430 and 928 expressions, respectively. Similar to the MOT Challenge setting [40], the test set contains both scenes that are similar to those in the training set and scenes that are entirely unseen during training. Figure 1 shows representative examples from Refer-ASV. The numbers displayed above the bounding boxes denote the object identity (ID) assigned by the tracker, which indicates the temporal association of the same object across frames. Table 2 summarizes the main statistics of the proposed Refer-ASV dataset.

#### 3.1.3. Dataset Statistics

Table 3 compares Refer-ASV with several representative RMOT datasets. Compared with existing datasets, Refer-ASV focuses on water-surface scenes and includes explicit environmental descriptions and fine-grained category annotations. In addition, Refer-ASV provides a larger number of expressions and a more diverse vocabulary.

**Figure 2:**
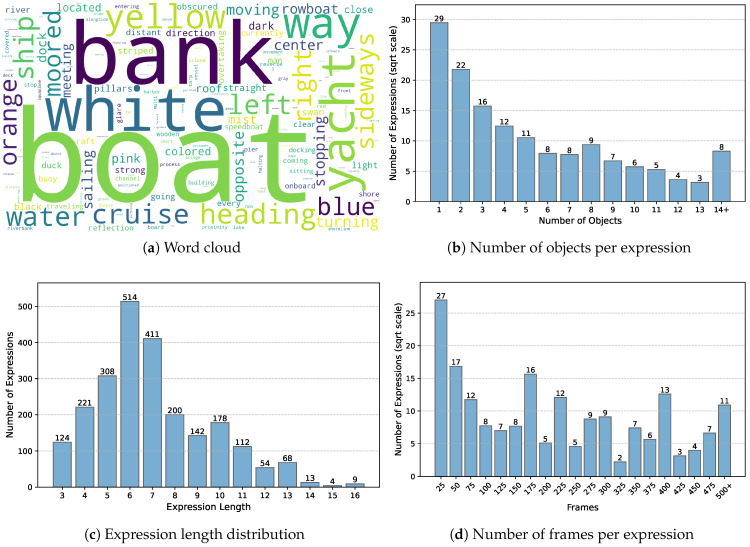
Data distributions of Refer-ASV.

To provide a more intuitive view of the dataset characteristics, Figure 2 summarizes the distributions of Refer-ASV from four aspects. As shown in Figure 2a, the vocabulary exhibits clear water-surface characteristics, with frequent references to fine-grained categories, color attributes, as well as water-specific actions and spatial location descriptions. Figure 2b shows that expressions referring to one or two objects constitute the majority, while a substantial portion involves multiple objects or dense object groups, indicating a wide range of referring granularities. The distribution of expression length in Figure 2c indicates that most expressions contain approximately 6–7 words, while a non-negligible fraction of longer expressions reflects increased linguistic complexity. Moreover, the temporal distribution in Figure 2d shows that expressions spanning fewer than 25 frames are dominant, mainly because certain behaviors are short-lived and the dataset includes expressions without referred objects. Longer frame spans are also well represented, with a notable number of expressions covering more than 500 frames. Overall, these statistics highlight the semantic diversity, referential flexibility, linguistic variability, and temporal coverage of Refer-ASV.

### 3.2. Methods

In this subsection, we present RAMOT, a water-surface-oriented RMOT method. We begin with a brief review of MOTR [22] and TempRMOT [29] as preliminaries, followed by an overview of the overall architecture. Building on this framework, we describe the semantic progressive expression sampling strategy and the scale-aware multimodal fusion mechanism. We then detail the query management strategy designed to maintain high-value tracklets.

#### 3.2.1. Preliminaries

**(1) MOTR** is a Transformer-based end-to-end MOT framework that unifies object detection and association within a single network. Instead of following the track-by-detection paradigm, MOTR jointly performs object localization and identity preservation through a query-based formulation. It maintains two types of queries: detect queries for identifying newly appearing objects, and track queries that represent complete tracklets. The latter are propagated and updated across frames. To support stable cross-frame association, MOTR adopts tracklet-aware label assignment and temporal feature aggregation, enabling temporal modeling without relying on additional association steps.

**(2) TempRMOT** is an end-to-end RMOT framework built upon MOTR. It introduces a temporal enhancement module (Temp.Enhance) to improve the temporal stability of object queries. Specifically, TempRMOT maintains a query memory that stores recent query features for each object. In the current frame, object queries interact with their historical representations through cross-frame attention in a temporal decoder, where temporal positional encoding is applied to model continuous motion and short-term history. The enhanced queries then participate in object-level interactions within the current frame and are used to predict residual updates for tracking results. By explicitly incorporating short-term temporal information, TempRMOT improves tracking stability under challenging conditions such as occlusion, fast motion, and appearance similarity.

#### 3.2.2. Overview

**Figure 3:**
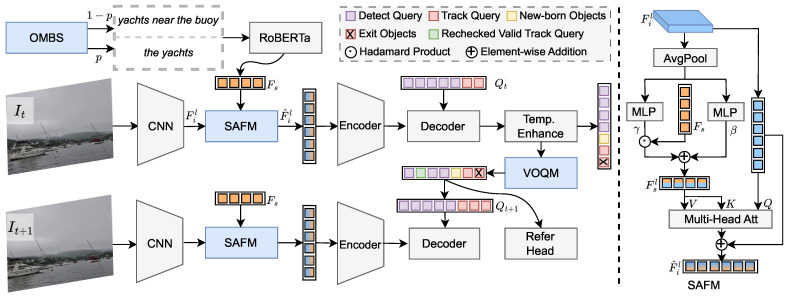
Overall architecture of RAMOT. During training, OMBS is used for expression sampling, while during inference, VOQM manages track queries and generates next-frame queries.

Our goal was to develop an end-to-end tracker for water-surface RMOT under complex scene conditions. To this end, we propose RAMOT, whose overall framework is illustrated in Figure 3. Formally, given a video sequence $V={\left\{I_{t}\right\}}_{t=1}^{T}$ and a referring expression *e*, the frame-wise inference process of RAMOT can be written as

(2) $${\hat{R}}_{t},\;Q_{t+1}=f_{\theta }\left(I_{t},e,Q_{t}\right),\;t=1,\ldots ,T,$$

where $Q_{t}$ refers to the query set at frame *t*, including detection queries and track queries, ${\hat{R}}_{t}$ denotes the prediction results at frame *t*, and $Q_{t+1}$ represents the updated queries propagated to the next frame.

Specifically, RAMOT follows the Transformer-based tracking framework of MOTR and incorporates three dedicated components: the one-more-boat sampling (OMBS) strategy, the scale-semantic-aware fusion module (SAFM), and the value-aware object query management (VOQM). These components enhance visual–language alignment at the training, feature representation, and inference stages, respectively. Taking two consecutive frames, $I_{t}$ and $I_{t+1}$, as illustrative inputs, during training, natural language expressions are generated by OMBS, while during testing, expressions are randomly sampled from the test set. A CNN backbone, ResNet50 [42], extracts multi-scale visual features $F_{i}^{l},l\in \left\{1\ldots L\right\}$, where *l* denotes the scale index and *L* is the total number of scales. In parallel, a frozen text encoder, RoBERTa [43], extracts referring features $F_{s}$ from the input expression. The visual features and referring features are then adaptively fused by SAFM to produce fused representations ${\hat{F}}_{i}^{l}$, which are fed into the encoder of Deformable DETR [44]. The encoder outputs, together with the query set $Q_{t}$ of the current frame consisting of detection queries and track queries, are passed to the decoder. The decoder outputs are processed by a Refer Head to produce initial predictions. The Refer Head adopts a multi-branch design, including a box branch for bounding-box regression, a class branch for empty-object identification, and a referring branch for determining whether an object matches the input expression. All branches are implemented using multilayer perceptrons (MLPs). Finally, the decoder outputs and initial predictions are refined by the Temp.Enhance module, which employs a memory mechanism to improve temporal consistency and capture object motion patterns, producing updated queries $Q_{t+1}$ during training and the final predictions during inference.

In addition, during the inference stage, VOQM selectively recovers missing but high-value objects to complement the outputs of the Temp.Enhance module, generating updated queries $Q_{t+1}$ for inference and producing the final tracklet predictions.

#### 3.2.3. One-More-Boat Sample Strategy

During RMOT training, the referring expression determines how many object tracklets provide supervision in each iteration. Most existing RMOT methods adopt random expression sampling, treating expressions with different semantic complexities as equally suitable throughout the training process. However, in water-surface scenes, RMOT expressions often involve fine-grained semantic distinctions across object categories, motion states, and trajectory context. This is mainly due to the small object scales, high visual similarity among vessels with fine-grained categories, weak motion constraints, and frequent object–environment interactions. The statistics in Section 3.1.3 further confirm the substantial diversity in expression complexity. In addition, because water-surface scenes are largely absent from pretraining datasets, models exhibit limited discriminative capability at the early stage of training. Consequently, introducing semantically complex expressions too early leads to sparse and unreliable supervision, resulting in unstable cross-modal alignment and optimization.

**Figure 4:**
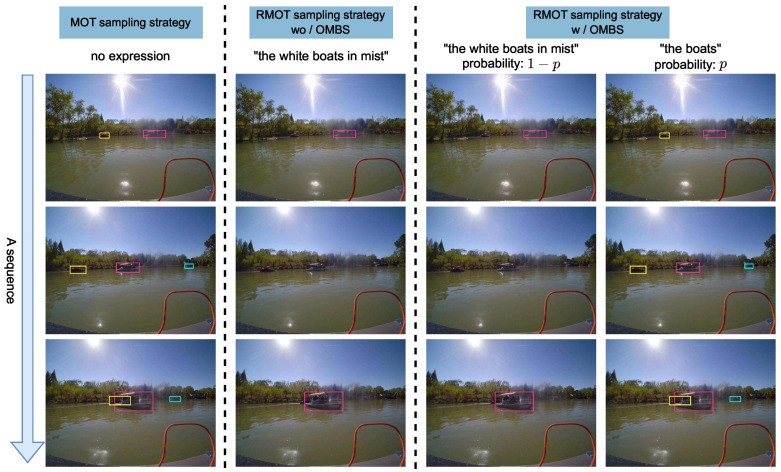
Comparison of different sampling strategies. Different colors denote different tracklets. (**Left**) MOT without expressions. (**Middle**) existing RMOT sampling. (**Right**) OMBS with low-ambiguity expression sampling.

To address these issues, we propose the OMBS strategy, which stabilizes early training by controlling the semantic granularity of referring expressions. In Refer-ASV, full referring expressions typically combine multiple semantic phrases, whereas the auxiliary expressions used in OMBS retain only the category-level phrase and therefore contain less semantic information. A comparison between OMBS, sampling strategies in MOT, and those used in existing RMOT methods is illustrated in Figure 4. Unlike uniform sampling over all expressions, OMBS organizes language supervision in a curriculum manner according to semantic complexity. At the early training stage, expressions with low ambiguity and limited semantic composition are preferentially introduced. This design reduces optimization difficulty and suppresses noisy supervision, enabling the model to establish stable visual–language alignment. As training progresses, expressions with finer granularity and more complex semantic compositions are gradually incorporated. By explicitly shaping the distribution of referring expressions over training, OMBS introduces curriculum learning [12] into the language supervision process.

Specifically, we construct an auxiliary expression set $E_{\mathrm{extra}}$ that contains only category-level descriptions of water-surface objects, such as “the boats”, “the yachts”, and “the buoys”. Among them, “the boats” serves as a generalized vessel category that covers all vessel instances and provides denser supervision at the early training stage. During training, expressions are sampled from $E_{\mathrm{extra}}$ with a time-varying probability p(t) and from the original expression set $E_{\mathrm{origin}}$ with probability 1−p(t). The OMBS process is formulated as

(3) $$x∼\left\{\begin{array}{cc} E_{\mathrm{extra}}, & \mathrm{with}\;\mathrm{prob}.p\left(t\right)=\max\left(0,\;p_{0}-\frac{t}{\Delta }\cdot \delta \right),\\ E_{\mathrm{origin}}, & \mathrm{with}\;\mathrm{prob}.1-p(t), \end{array}\right.$$

where $p_{0}$ denotes the initial sampling probability, Δ is the epoch interval controlling probability decay, and δ is the decay step. As training progresses, the sampling gradually shifts toward $E_{\mathrm{origin}}$, exposing the model to increasingly specific and semantically complex expressions and enabling a transition from coarse-grained semantic alignment to fine-grained referring understanding.

#### 3.2.4. Scale-Semantic-Aware Fusion Module

In existing RMOT frameworks, visual–language alignment is typically performed over multi-scale visual features. However, visual features at different scales encode semantics at different levels and thus play distinct roles [45]. High-resolution features emphasize fine structural details and are crucial for accurate localization of small objects, whereas low-resolution features mainly capture more abstract semantics, such as object categories and contextual information. Most RMOT methods align shared language features with visual features at all scales, implicitly treating language semantics as scale-agnostic. In water-surface scenes, where small objects are prevalent and often described by fine-grained semantic attributes, this uniform alignment can lead to mismatches between language cues and visual representations across scales.

To address this issue, we propose the SAFM module, which performs scale-specific language–visual alignment by conditionally modulating language features with visual context at each scale. Its structure is illustrated in the right part of Figure 3. For the visual feature $F_{i}^{l}$ at the *l*th scale, we first extract global semantic information via global average pooling. The pooled representation is then fed into two lightweight MLPs to generate scale-dependent modulation parameters $\left(\gamma^{l},\beta^{l}\right)$:

(4) $$\left(\gamma^{l},\beta^{l}\right)=\mathrm{MLP}\left(\mathrm{AvgPool}\left(F_{i}^{l}\right)\right),l\in \left\{1,\ldots ,L\right\}.$$

These parameters are used to modulate the language feature $F_{s}$ produced by the text encoder, yielding scale-conditioned language features $F_{s}^{l}$:

(5) $$F_{s}^{l}=\gamma^{l}⊙F_{s}+\beta^{l}.$$

The conditioned language features are then fused with the corresponding visual features at each scale through multi-head cross-attention, producing scale-specific multimodal representations ${\hat{F}}_{i}^{l}$:

(6) $$Q=\mathrm{Proj}\left(F_{i}^{l}+P_{V}\right),K=\mathrm{Proj}\left(F_{s}^{l}+P_{S}\right),V=F_{s}^{l},$$

(7) $${\hat{F}}_{i}^{l}=F_{i}^{l}+\frac{QK^{T}}{\sqrt{d}}V.$$

Here, Proj denotes a linear projection implemented by an MLP, $P_{V}$ and $P_{S}$ are the positional encodings for visual and textual features [46,47], respectively, and *d* is the feature dimension of the QKV representations.

#### 3.2.5. Value-Aware Object Query Management

**Figure 5:**
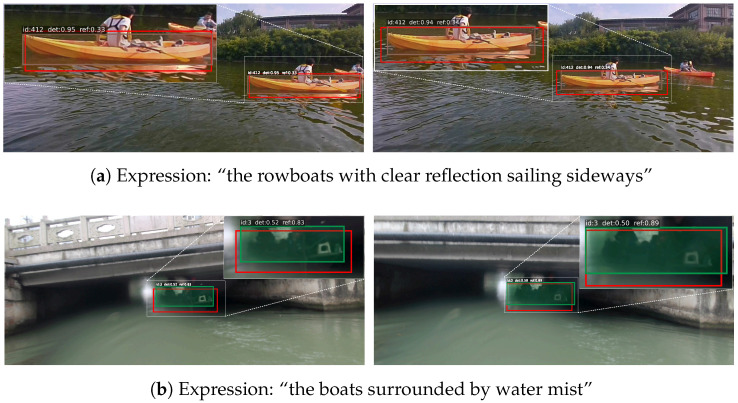
Examples of ambiguous query activation under challenging water-surface scenes. Red boxes denote ground truth, and boxes in other colors denote predictions. The values above each predicted box indicate the object ID, detection confidence, and referring confidence.

During inference, existing end-to-end RMOT methods typically follow the fixed query management strategy inherited from MOTR. In this strategy, query activation and maintenance are jointly determined by detection confidence and referring confidence, using fixed thresholds to decide whether to initialize a new trajectory, retain an existing one, or terminate a track. Such a threshold-based strategy is effective in semantically clear settings, such as MOT with predefined object categories or RMOT with simple expressions under favorable imaging conditions. However, water-surface scenes often involve substantial uncertainty, under which detection confidence and referring confidence may become misaligned. This misalignment is particularly pronounced at the onset of complex motions or under degraded imaging conditions, such as fog and water spray. For example, at the early stage of a complex action, an object may exhibit high detection confidence while its referring semantics have not yet been sufficiently established, resulting in low referring confidence (Figure 5a). Conversely, under degraded imaging conditions, an object may maintain high referring confidence while detection confidence is temporarily suppressed (Figure 5b). In these cases, existing query management strategies may prematurely discard potentially valid and critical object queries.

To mitigate this issue, we propose VOQM, which introduces a short-term window-based re-evaluation mechanism to temporarily buffer and reassess object queries that satisfy complementary confidence conditions. Specifically, if an object meets either of the following criteria, (1) detection confidence $>\tau_{d}^{+}$ and referring confidence $>\tau_{r}^{-}$ (Condition 1), or (2) referring confidence $>\tau_{r}^{+}$ and detection confidence $>\tau_{d}^{-}$ (Condition 2), the corresponding query is placed into a pre-activation pool and re-evaluated over a short temporal window. The thresholds are determined according to the score distributions of detection confidence and referring scores and are selected to balance false positives and false negatives during query filtering. Formally, let $s_{d,t}^{i}$ and $s_{r,t}^{i}$ denote the detection confidence and referring confidence of the *i*th query at frame *t*, respectively. The pre-activation condition can be written as

(8) $$q_{t}^{i}\in P_{t}\Leftrightarrow \left(s_{d,t}^{i}>\tau_{d}^{+}\wedge s_{r,t}^{i}>\tau_{r}^{-}\right)\;\vee \;\left(s_{r,t}^{i}>\tau_{r}^{+}\wedge s_{d,t}^{i}>\tau_{d}^{-}\right),$$

where $P_{t}$ denotes the pre-activation pool at frame *t*. A query is activated as a valid trajectory only if it remains stable across consecutive frames, which is formulated as

(9) $$q_{t}^{i}\in A_{t}\Leftrightarrow q_{t}^{i}\in P_{t}\wedge q_{t-1}^{i}\in P_{t-1},$$

where $A_{t}$ denotes the set of activated queries at frame *t*. By deferring irreversible activation decisions when visual or semantic cues are temporarily unreliable, VOQM reduces the risk of missing high-value objects under challenging conditions.

## 4. Results

In this section, we report the results of experiments conducted to analyze the characteristics of water-surface RMOT and to evaluate the effectiveness of the proposed baseline method, RAMOT. We first introduce the datasets and evaluation metrics used for assessment, followed by the implementation details. We then benchmark publicly available RMOT methods on the constructed Refer-ASV dataset and evaluate cross-scene generalization on Refer-KITTI [10]. Ablation studies are then presented to analyze the contribution of individual components. Finally, representative prediction results are also visualized for a qualitative analysis.

### 4.1. Datasets and Metrics

**(1) Datasets.** To evaluate performance and cross-scene generalization, we conducted experiments on both Refer-ASV and Refer-KITTI. Refer-ASV is the dataset proposed in this work, which focuses on referring multi-object tracking in water-surface navigation scenes. Refer-KITTI is derived from the KITTI dataset [33] and focuses on ground-level driving scenes captured from onboard cameras. It contains 18 video sequences annotated with 818 referring expressions, where expressions describe vehicles or pedestrians using appearance, spatial position, and motion cues.

**(2) Evaluation metrics.** We adopted higher-order tracking accuracy (HOTA) [48] as the primary evaluation metric for overall tracking performance. HOTA is a unified metric that jointly evaluates detection quality and association quality, providing a more balanced assessment than earlier metrics such as MOTA [49] or IDF1 [50]. It is defined based on detection and association scores computed at different localization thresholds α:

(10) $${\mathrm{HOTA}}_{\alpha }=\sqrt{{\mathrm{DetA}}_{\alpha }\cdot {\mathrm{AssA}}_{\alpha }},$$

where DetAα and ${\mathrm{AssA}}_{\alpha }$ denote the detection accuracy and association accuracy at threshold α, respectively. Detection accuracy DetAα measures the consistency between predicted and ground-truth detections by accounting for true positives (TP), false positives (FP), and false negatives (FN):

(11) $${\mathrm{DetA}}_{\alpha }=\frac{|{\mathrm{TP}}_{\alpha }|}{|{\mathrm{TP}}_{\alpha }\left|+\right|{\mathrm{FP}}_{\alpha }\left|+\right|{\mathrm{FN}}_{\alpha }|}.$$

Association accuracy AssAα evaluates identity consistency for matched detections and is defined as

(12) $${\mathrm{AssA}}_{\alpha }=\frac{{\mathrm{AssRe}}_{\alpha }\cdot {\mathrm{AssPr}}_{\alpha }}{{\mathrm{AssRe}}_{\alpha }+{\mathrm{AssPr}}_{\alpha }-{\mathrm{AssRe}}_{\alpha }\cdot {\mathrm{AssPr}}_{\alpha }},$$

where AssReα and AssPrα denote the recall and precision of association, respectively. In practice, HOTA is averaged over a range of localization thresholds to obtain a single overall tracking score. By jointly accounting for detection accuracy and identity association, HOTA provides a comprehensive evaluation of both localization quality and identity consistency over time.

### 4.2. Implementation Details

During training, the convolutional backbone, encoder, decoder, and the detection and classification branches of the Refer Head were initialized with the official pretrained weights of Deformable DETR [44]. The text encoder adopted the official RoBERTa [43] implementation and pretrained weights, and remained frozen throughout training, following existing MOTR-based RMOT frameworks. All remaining parameters were randomly initialized. We employed the multi-branch data augmentation strategy from MOTR, including multi-scale resizing and cropping, followed by normalization. Temporal modeling used a clip sampling strategy with a clip length of five frames and a stride of one, following the default configuration of MOTR-based tracking frameworks. During inference, only standard resizing and normalization were applied. Training followed the loss formulation in [29], jointly optimizing classification loss (focal loss [51]), bounding-box regression losses (L1 and GIoU [52]), and referring loss (focal loss) in an end-to-end manner. The model was trained for 60 epochs using the Adam optimizer, with an initial learning rate of $1\times 10^{-5}$ for the backbone and $1\times 10^{-4}$ for all other parameters. The learning rate was decayed by a factor of 10 at epoch 40 and subsequently scheduled using a StepLR policy. OMBS was applied only during training, with the initial sampling probability $p_{0}$ set to 0.2 and both the decay interval Δ and decay step δ set to five. VOQM was applied only during inference, with $\tau_{d}^{+}$, $\tau_{r}^{-}$, $\tau_{r}^{+}$, and $\tau_{d}^{-}$ set to 0.8, 0.3, 0.7, and 0.5, respectively. All experiments were conducted on four NVIDIA RTX 3090 GPUs with a batch size of one per GPU. Identical training and evaluation settings were used across all experiments to ensure fair comparison and reproducibility. Table 4 summarizes the main software and hardware environment used in this work.

### 4.3. Comparison with State of Art

In this subsection, we benchmark representative RMOT methods on Refer-ASV, including publicly available approaches and our method. We further evaluate the generalization of our method to other scenes. Specifically, we introduce the evaluated methods and report quantitative results on both Refer-ASV and Refer-KITTI.

#### 4.3.1. Evaluated Methods

The evaluated methods are summarized below. For fair comparison, all publicly available models were trained and tested on Refer-ASV using the settings from their official implementations. We focused on dedicated RMOT methods rather than adapting water-surface MOT baselines (e.g., Sea-IoU Tracker and VTM-YS) through simple early visual–language fusion, as previous benchmarks indicated that specialized RMOT architectures consistently outperformed such adapted baselines on referring tasks [10]. DeepRMOT [53] and CDRMT [31] are not publicly available; therefore, we directly report their results on Refer-KITTI as provided in the original papers.

1. **iKUN** [32]: A two-stage RMOT framework that decouples tracking and language grounding through an insertable knowledge unification module, enabling referring tracking with frozen off-the-shelf trackers.
2. **TransRMOT** [10]: The first end-to-end Transformer-based RMOT framework, which integrates early visual–language fusion with decoupled detection and track queries to track objects referred to by natural language expressions.
3. **TempRMOT** [29]: A query-based temporal enhancement framework that aggregates historical query features via a memory mechanism to improve temporal modeling and association robustness.
4. **MGLT** [30]: A multigranularity localization Transformer that mitigates linguistic cue attenuation by bootstrapping text-aware track queries and enforcing dense track-prompt alignment for referring localization and association.
5. **DeepRMOT**: A deep cross-modal fusion framework that combines early fusion, bidirectional vision–language encoding, and cross-modal decoding to enhance visual–linguistic representations for detection and association.
6. **CDRMT**: A contrastive-driven RMOT framework that jointly optimizes visual–language representation learning and data association through cross-modal contrastive constraints to improve robustness under semantic ambiguity.

#### 4.3.2. Evaluation on the Refer-ASV Dataset

Table 5 reports the performance comparison of several publicly available RMOT methods and our approach on Refer-ASV. RAMOT achieves a HOTA score of 39.97, ranking first among all evaluated methods. It outperforms TransRMOT, TempRMOT, and MGLT by 6.58 (19.7%), 3.76 (10.38%), and 3.79 (10.48%), respectively. Compared with iKUN, RAMOT obtains a larger HOTA gain of 11.62 (40.99%), suggesting that two-stage frameworks are less suited to complex water-surface scenarios. Moreover, RAMOT also achieves the best performance on the two primary components of HOTA, namely, DetA and AssA, with scores of 23.55 and 70.48, respectively. In Refer-ASV, objects are often small in scale, vessel appearances are highly similar with fine-grained category distinctions, and scenes are frequently affected by water reflections, waves, and illumination variations. These factors jointly increase the difficulty of both object detection and trajectory association. The same-direction comparison with road traffic scenes in Table 6 further reflects the challenging nature of Refer-ASV.

Specifically, different methods exhibit distinct failure patterns in water-surface scenes. TransRMOT maintains relatively high precision-related scores, but its DetA and AssA are notably lower, indicating frequent missed detections or premature track termination at the onset of motion or under degraded imaging conditions. TempRMOT and MGLT improve AssA by introducing temporal modeling or language-aware designs; however, their DetA remains limited, reflecting insufficient capability in recognizing small-scale and low-contrast water-surface objects. In contrast, RAMOT achieves consistent improvements in both DetA and AssA, indicating that more described objects are successfully detected and continuously tracked under complex water-surface conditions, leading to higher overall HOTA performance.

To further examine robustness under different water-surface conditions, we also report condition-wise results on Refer-ASV. Table 7 presents the tracking performance under four environmental conditions, including sunny, cloudy, mist, and strong-light scenes. RAMOT achieves the best HOTA in all settings. The improvement is more evident in challenging scenes. In mist scenes, RAMOT improves HOTA from 20.74 to 23.44 compared with TempRMOT. In strong-light scenes, the improvement increases from 24.19 to 28.52. These results indicate that the proposed method remains more stable when visibility is reduced or illumination is unstable. RAMOT also shows consistent gains in sunny and cloudy scenes, suggesting that the improvement is not limited to specific environmental conditions.

#### 4.3.3. Generalization Analysis

To further evaluate the generalization ability of RAMOT, we conducted experiments on Refer-KITTI, as reported in Table 6. All methods achieved substantially higher performance on Refer-KITTI than on Refer-ASV, which reflects the comparatively lower difficulty of RMOT in road-scene settings. On that dataset, RAMOT attained a HOTA of 52.94, a DetA of 40.98, and an AssA of 68.65, achieving the best overall performance. Compared with Refer-ASV, the relative performance gains of RAMOT on Refer-KITTI were more limited, which could be attributed to differences in dataset characteristics. A direct train-on-one and test-on-the-other transfer setting would not cleanly isolate scene-domain differences, because Refer-KITTI and Refer-ASV differ not only in scene characteristics but also in object categories and expression semantics. First, Refer-KITTI contains only two object categories, namely, vehicles and pedestrians, resulting in lower semantic complexity and ambiguity in referring expressions. This constrains the potential benefits of OMBS and VOQM, which are designed to handle complex and uncertain language supervision. Second, road scenes exhibit a narrower range of object scales and more constrained motion patterns than water-surface scenes, which reduces the advantage of SAFM. Despite these factors, RAMOT still achieved consistent improvements in both DetA and AssA, indicating that the proposed design generalizes across datasets rather than being tailored exclusively to water-surface scenes.

### 4.4. Ablation Study

In this subsection, we analyze the contribution of each component in RAMOT on Refer-ASV and thoroughly examine the impact of different settings.

#### 4.4.1. Ablation of the Proposed Components

To verify the effectiveness of individual components in RAMOT, we conducted ablation studies on the Refer-ASV test set, as reported in Table 8. The baseline model without OMBS, SAFM, or VOQM achieved a HOTA of 37.95, with DetA and AssA of 21.79 and 68.42, respectively. Introducing OMBS alone increased HOTA to 38.34, mainly due to a 0.70 gain in DetA, while AssA showed a slight decrease. This suggests that curriculum-based expression sampling helps the model detect more relevant objects during early training but offers limited benefit to trajectory association when used in isolation. When SAFM was applied alone, HOTA rose to 38.57 and DetA improved to 22.55, whereas AssA remained nearly unchanged, indicating that scale-aware semantic alignment primarily benefits object localization. When OMBS and SAFM were jointly applied, HOTA further increased to 39.23, with DetA and AssA reaching 23.08 and 69.43, respectively. This result shows that stable language supervision and scale-aware feature alignment are complementary, contributing to both object localization and identity maintenance. Finally, incorporating all three components (OMBS, SAFM, and VOQM) yielded the best overall performance, with HOTA improving to 39.97 and DetA and AssA reaching 23.55 and 70.48, respectively. Notably, AssA exhibited the largest gain, indicating that short-term buffering and recheck of queries can recover missing yet critical objects under complex water-surface imaging conditions, thereby reducing the missing tracklets caused by unstable confidence signals.

To further examine the efficiency of the proposed modules, we additionally report the computational cost, inference speed, and tracking performance of different component settings in Table 9. Compared with the base model, adding SAFM increases the total parameter count from 174.51 M to 175.04 M, while the trainable parameters increase from 49.64 M to 50.17 M. Meanwhile, the FLOPs change only from 237.61 G to 237.62 G, and the FPS decreases slightly from 12.78 to 12.51. In return, HOTA improves from 38.34 to 39.23. After further adding VOQM, the computational cost remains unchanged, and the speed stays stable at 12.56 FPS, while HOTA further improves to 39.97. The result shows that the proposed modules improve tracking accuracy with only marginal extra cost. It is worth noting that existing end-to-end RMOT frameworks already include multi-scale features and cross-attention operations. SAFM does not introduce a new heavy multi-scale fusion branch. Instead, it only adds lightweight MLP layers to generate scale-dependent modulation parameters. Therefore, the additional overhead of SAFM is limited. Moreover, ASV scenes usually involve smoother short-term target motion than road scenes, so the current inference speed remains suitable for online use in our setting.

**Figure 6:**
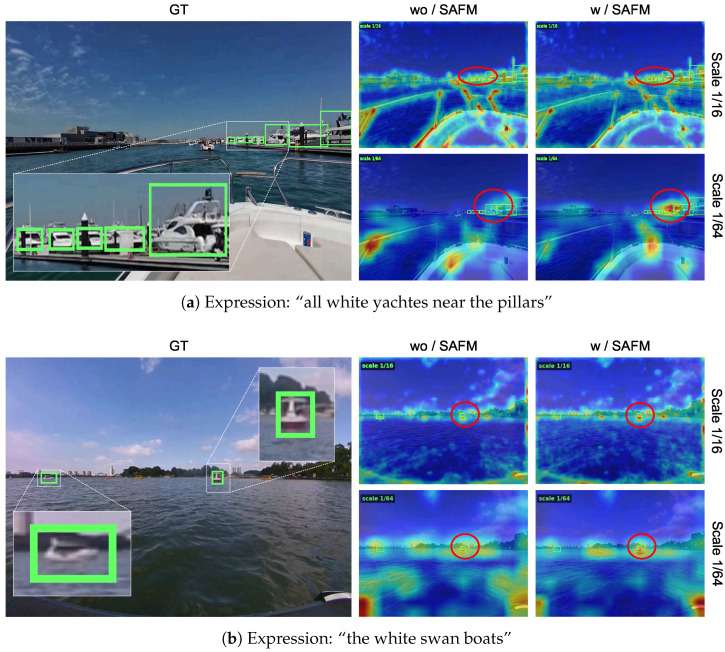
Scale-wise activation visualization of SAFM. Red circles highlight regions with clear response differences. Green boxes denote ground-truth objects. Best viewed when zoomed in.

To better understand how SAFM affects localization across scales, we provide a scale-wise activation visualization in Figure 6. Two representative examples are shown. In each example, we compare feature responses without and with SAFM at two representative scales, namely 1/16 and 1/64. After SAFM is introduced, the responses around referred objects become more concentrated. The effect is especially clear for small objects. In Figure 6a, SAFM strengthens the response of a small object at the 1/16 scale and enhances the response of a relatively larger referred object at the 1/64 scale. In Figure 6b, the responses of small referred objects are strengthened at both scales. These results support that SAFM improves scale-specific visual–language alignment and benefits object localization in water-surface RMOT.

#### 4.4.2. Ablation of OMBS

**Figure 7:**
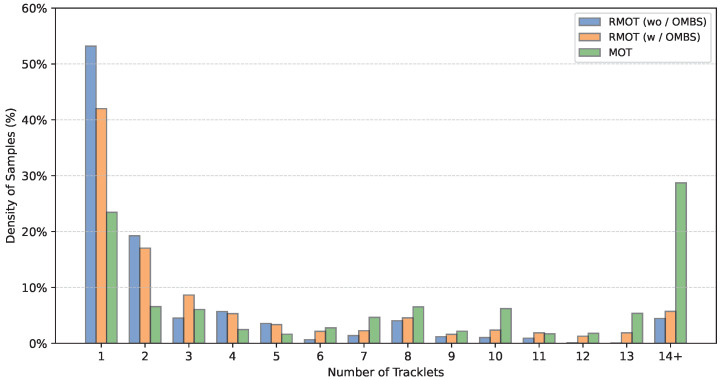
Distribution of the number of tracklets in sampled instances at the first training epoch. “w/o OMBS” denotes the sampling strategy used in existing RMOT methods, and “w/ OMBS” denotes the OMBS strategy.

In this subsection, we investigate the effect of OMBS and analyze the influence of different parameter settings. Figure 7 shows the distribution of the number of tracklets in sampled instances at the first training epoch. Introducing OMBS increases the number of tracklets covered by sampled expressions at the early stage, with a clear rise in the proportion of samples containing six or more tracklets. This is because OMBS introduces low-ambiguity expressions with coarse semantic granularity during early training, which typically correspond to a larger set of tracklets. By reshaping the distribution of sampled expressions, OMBS provides denser and more stable supervision at the beginning of training.

Table 10 reports the ablation results under different OMBS parameter settings on Refer-ASV. When $p_{0}=0.2$, Δ=5, and δ=5, the model achieves the best performance, with HOTA, DetA, and AssA reaching 39.97, 23.55, and 70.48, respectively. When the decay is too fast, such as Δ=1 and δ=1, the sampling probability rapidly shifts from $E_{\mathrm{extra}}$ to $E_{\mathrm{origin}}$, causing the model to encounter more semantically complex expressions at an early stage. In water-surface scenes, where pretrained priors are limited, this leads to sparse and unstable supervision and results in noticeable drops in both DetA and AssA. In contrast, when the decay is too slow, such as Δ=20 or Δ=30, DetA decreases markedly, indicating that prolonged reliance on coarse expressions hinders learning of fine-grained semantics.

The effect of the initial sampling probability $p_{0}$ further highlights the importance of balancing semantic supervision. When $p_{0}=0.1$, HOTA drops to 36.41, as early supervision from $E_{\mathrm{extra}}$ is insufficient, and the model is forced to handle semantically complex expressions before stable visual–language alignment is established. With Δ and δ fixed, increasing $p_{0}$ strengthens dense supervision at early training stages but reduces both the probability and duration of exposure to fine-grained expressions (Rows 2 and 5). All these observations indicate that OMBS benefits from a moderate initial sampling probability and a gradual decay schedule, which facilitates a smooth transition from coarse-grained semantic alignment to fine-grained referring understanding, thereby improving overall tracking performance.

#### 4.4.3. Ablation of VOQM

In this subsection, we analyze the effect of VOQM on Refer-ASV. Table 11 examines the impact of different VOQM configurations, while Table 12 and Table 13 report ablation results under different parameter settings. Following the threshold settings adopted in existing end-to-end RMOT methods [10,29,30], the initial object filtering used a detection threshold of 0.6 and a referring threshold of 0.4. When VOQM was enabled, an additional recheck step was applied after the initial filtering, including both HDLR and HRLD mechanisms.

**(1)**  **Effect of different VOQM configurations.**

As shown in Table 11, without VOQM, the model achieves a HOTA of 39.23, with DetA and AssA of 23.08 and 69.43, respectively, relying solely on the initial object filtering strategy. Enabling HDLR, which re-evaluates objects with high detection confidence but low referring confidence, increases HOTA to 39.56 and AssA to 70.14. This indicates that at the early stage of complex actions or when referring semantics are not yet stable, objects supported by reliable visual evidence can be retained. When HRLD is enabled, which re-evaluates objects with high referring confidence but low detection confidence, HOTA further rises to 39.87 and AssA to 70.75. This suggests that under degraded imaging conditions, such as reflections and water spray, objects with strong semantic consistency but temporarily suppressed detection confidence can be recovered. When both HDLR and HRLD are applied, the model achieves the best overall performance, with HOTA, DetA, and AssA reaching 39.97, 23.55, and 70.48, respectively. This result shows that the two re-evaluation conditions are complementary and jointly mitigate track loss caused by short-term confidence mismatch in water-surface scenes.

**(2)**  **Analysis of different VOQM parameter settings.**

Table 12 reports the ablation results under different HDLR parameter settings. HDLR rechecks objects with high detection confidence but low referring confidence, a situation that commonly occurs at the early stage of complex actions that require longer-term semantic confirmation. When $\tau_{d}^{+}$ is set equal to the initial detection threshold (0.6), only $\tau_{r}^{-}=0.3$ takes effect, resulting in limited improvement compared with the setting without HDLR. With $\tau_{r}^{-}$ fixed, increasing $\tau_{d}^{+}$ to 0.8 raises HOTA from 39.47 to 39.56 and AssA from 69.97 to 70.14. This reflects that when semantic cues are unstable, relying on more confident visual evidence is beneficial, whereas a lower detection threshold may introduce poorly localized objects and increase false positives. With $\tau_{d}^{+}$ fixed, reducing $\tau_{r}^{-}$ to 0.2 leads to decreases in both HOTA and AssA, as objects with highly ambiguous semantics are introduced and degrade association quality. In contrast, a moderate $\tau_{r}^{-}$ allows temporary fluctuations in referring confidence, resulting in more robust tracking.

Table 13 presents the ablation results under different HRLD parameter settings. HRLD rechecks objects with high referring confidence but low detection confidence, which frequently occurs under degraded imaging conditions such as water reflections, water spray, and strong illumination. As shown in the table, a higher $\tau_{r}^{+}$ helps retain objects that are highly consistent with the referring semantics. Setting $\tau_{d}^{-}$ slightly below the initial detection threshold (0.6) enables the recovery of high-value objects while avoiding candidates with extremely low detection confidence. In practice, the setting $\tau_{r}^{+}=0.7$ and $\tau_{d}^{-}=0.5$ effectively recovers trajectories temporarily interrupted by short-term detection degradation, thereby improving overall tracking performance. These results provide empirical evidence that the short-term re-evaluation is constrained rather than self-reinforcing: loosening the recheck thresholds does not lead to continuous gains but instead may introduce ambiguous queries and degrade tracking quality.

### 4.5. Visualization

**Figure 8:**
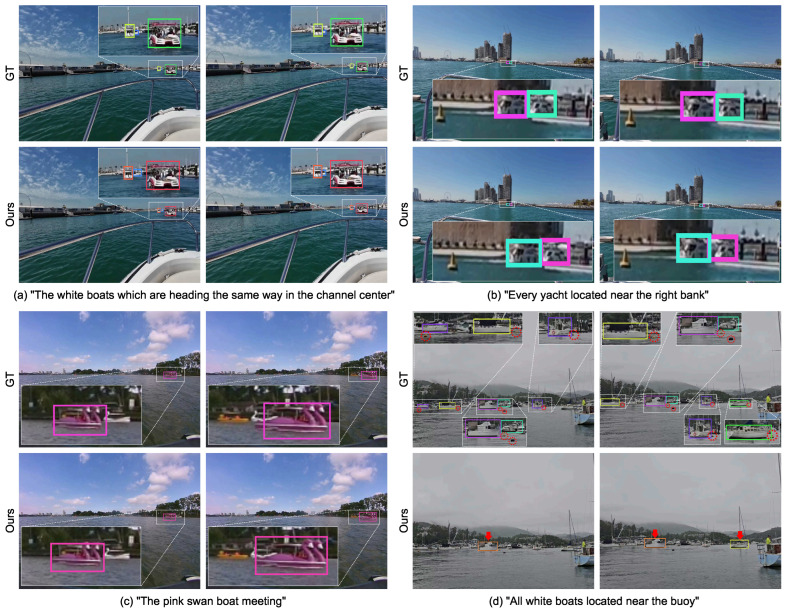
Representative tracking results of RAMOT on the Refer-ASV test set. Different colored boxes denote different trajectories. (**a**–**c**) show successful cases, and (**d**) shows a failure case, where the buoys are highlighted by red dashed outlines in the ground truth.

Figure 8 presents representative tracking results of RAMOT on the Refer-ASV test set. Cases (a), (b), and (c) illustrate successful cases under different challenging conditions. In (a), the referring expression is semantically complex, involving color attributes, actions relative to the ego vessel’s heading, and imprecise relative locations. This case requires accurate understanding of compound semantics and includes multi-scale objects as well as partial occlusions. Case (b) focuses on fine-grained categories with highly similar appearances, where all referred objects are small white vessels. Case (c) involves fine-grained categories and interaction behaviors with other objects.

Case (d) shows a failure case, in which the interacting objects described in the expression are extremely small buoys, and the model fails to track all of them consistently. Overall, these results indicate that the proposed approach achieves effective visual–language alignment and can reliably track fine-grained, semantically complex, and multi-scale objects specified by natural language expressions. However, interactions involving extremely small objects remain challenging, highlighting that water-surface RMOT is still a demanding problem and warrants further investigation.

## 5. Conclusions

This work investigated RMOT in water-surface scenes for ASV navigation. Unlike road scenes, water-surface environments are characterized by reflections, illumination variations, small object scales, and weakly constrained object motions. These factors affect not only visual tracking but also the way objects are described and referred to, making water-surface RMOT different from existing benchmarks. To study this problem in a realistic setting, we constructed Refer-ASV based on real ASV navigation videos. The dataset emphasized fine-grained vessel categories, water-specific behaviors, and environment-related expressions that frequently arise in practical operations. Based on this benchmark, we introduced RAMOT as an end-to-end baseline to enable consistent evaluation. Experimental results across different scenes showed that addressing these challenges is important for reliable language-guided tracking in water-surface environments. We expect Refer-ASV to serve as a useful testbed for future RMOT research and to encourage the development of methods better aligned with real-world water-surface navigation. Despite these contributions, constructing such a benchmark remains challenging due to the scarcity of shipborne data and the high cost of data collection, which also limits the coverage of certain conditions such as nighttime or extremely dark scenes. In future work, we plan to extend the dataset to more diverse environments and imaging conditions and to explore more robust tracking methods for extremely small objects, complex interactions, and multimodal water-surface perception scenes.

## Figures and Tables

**Table 1:** Basic templates used for expression generation.

Template	Example
the {*color*} {*category*}	the white duck boats
the {*color*} {*category*} with {*attribute*}	the orange boats with people onboard
the {*category*} {*action*}	the boats sailing sideways
the {*color*} {*category*} {*position*}	the yellow speedboats in the center of the channel
the {*category*} {*action*} {*environment*}	the boats sailing straight in water mist

**Table 2:** Statistics of the proposed Refer-ASV dataset.

Property	Value
Scene	Water surface
Sequences	29
Frames	10,747
Object classes	15
Referring expressions	2358
Vocabulary size	107
Average expression length (AEL)	6.93
Train frames/sequences	6761/15
Test frames/sequences	3986/14
Train/test expressions	1430/928

**Table 3:** Comparison of Refer-ASV with representative RMOT datasets.

Dataset	Scene	WE	WFC	Exps	Vocabs	AEL	Classes	Videos	Frames
Refer-KITTI [10]	road	✗	✗	818	49	4.62	2	18	6.6k
Refer-Dance [32]	dancing	✗	✗	1985	25	7.60	1	65	67.3k
RMOT-SV [36]	satellite	✗	✗	556	80	6.15	5	212	33.1k
Refer-ASV (ours)	water	✓	✓	2358	107	6.93	15	29	10.7k

**WE**: with environment descriptions; **WFC**: with fine-grained category descriptions; **Exps**: number of expressions; **Vocabs**: vocabulary size; **AEL**: average expression length (in words).

**Table 4:** Software and hardware environment used in this work.

Software/Tool	Version
Python	3.9
PyTorch	2.3
CUDA	12.6
GPU	NVIDIA RTX 3090
CPU	AMD EPYC 7302
LLM in expression expansion	Qwen3-Plus (API)

**Table 5:** Overall evaluation results of different RMOT methods on Refer-ASV. All values are reported before the percentage sign (%). The best results in each column are highlighted in **bold**, and the second-best results in each column are underlined.

Methods	HOTA	DetA	AssA	DetRe	DetPr	AssRe	AssPr	LocA
iKUN	28.35	14.73	56.84	19.92	30.87	60.12	84.37	78.76
TransRMOT	33.39	21.63	53.85	28.71	**43.89**	55.49	**90.57**	82.01
TempRMOT	36.21	20.56	65.98	28.13	40.84	69.90	85.67	**82.05**
MGLT	36.18	19.73	68.70	29.49	35.35	72.74	84.33	81.12
RAMOT (ours)	**39.97**	**23.55**	**70.48**	**32.67**	42.64	**74.70**	86.11	81.18

**Table 6:** Comparison of tracking performance between RAMOT and several state-of-the-art RMOT methods on Refer-KITTI. The best results in each column are highlighted in **bold**.

Methods	HOTA	DetA	AssA	DetRe	DetPr	AssRe	AssPr	LocA
iKUN	48.84	35.74	66.80	51.97	52.25	72.95	87.09	-
TransRMOT	45.15	37.54	54.56	49.88	58.66	57.61	**90.72**	90.08
TempRMOT	52.21	40.95	66.75	55.65	**59.25**	71.82	87.76	90.40
DeepRMOT	39.55	30.12	53.23	41.91	47.47	58.47	82.16	80.49
CDRMT	49.35	40.34	60.56	54.54	59.30	64.70	89.80	**90.61**
RAMOT (ours)	**52.94**	**40.98**	**68.65**	**56.96**	57.42	**74.06**	88.05	90.51

**Table 7:** HOTA comparison under different environmental conditions on Refer-ASV. The best results in each column are highlighted in **bold**.

Methods	Sunny	Cloudy	Mist	Strong Light	All
TransRMOT	34.41	29.09	11.92	20.79	33.39
TempRMOT	37.03	32.40	20.74	24.19	36.21
RAMOT (ours)	**40.76**	**36.55**	**23.44**	**28.52**	**39.97**

**Table 8:** Results of the ablation experiments of proposed components. All values are reported before the percentage sign (%). The best results in each column are highlighted in **bold**.

OMBS	SAFM	VOQM	HOTA	DetA	AssA
			37.95	21.79	68.42
✓			38.34	22.49	68.06
	✓		38.57	22.55	68.41
✓	✓		39.23	23.08	69.43
✓	✓	✓	**39.97**	**23.55**	**70.48**

**Table 9:** Computational cost and inference speed of different component settings. **Base** means only OMBS is applied. The numbers in parentheses indicate the number of trainable parameters.

Models	Params (M)	FLOPs (G)	FPS	HOTA
Base	174.51 (49.64)	237.61	**12.78**	38.34
Base + SAFM	175.04 (50.17)	237.62	12.51	39.23
Base + SAFM + VOQM	175.04 (50.17)	237.62	12.56	**39.97**

All FPS values were measured on a single NVIDIA RTX 3090.

**Table 10:** Ablation of different settings in OMBS. The best results in each column are highlighted in **bold**.

Init Prob $p_{0}$	Decay Epoch Δ	Decay Step δ	HOTA	DetA	AssA
**0.2**	**5**	**5**	**39.97**	**23.55**	**70.48**
0.2	1	1	38.61	22.74	68.06
0.2	20	20	38.25	21.59	70.20
0.3	10	10	38.28	22.75	67.00
0.3	1	1	38.13	21.90	69.06
0.3	30	30	38.02	21.85	68.64
0.1	10	10	36.41	20.58	66.57

**Table 11:** Ablation results of different VOQM configurations. The best results in each column are highlighted in **bold**.

HDLR	HRLD	HOTA	DetA	AssA
		39.23	23.08	69.43
✓		39.56	23.17	70.14
	✓	39.87	23.32	**70.75**
✓	✓	**39.97**	**23.55**	70.48

HDLR: recheck of objects with high detection confidence and low referring confidence.

**Table 12:** Ablation study of different **HDLR** settings. The best results in each column are highlighted in **bold**.

$\tau_{d}^{+}$	$\tau_{r}^{-}$	HOTA	DetA	AssA
0.6	0.3	39.47	23.12	69.97
0.8	0.2	39.40	23.15	69.63
**0.8**	**0.3**	**39.56**	**23.17**	**70.14**

**Table 13:** Ablation study of different **HRLD** settings. The best results in each column are highlighted in **bold**.

$\tau_{r}^{+}$	$\tau_{d}^{-}$	HOTA	DetA	AssA
0.4	0.5	39.70	23.28	70.27
0.7	0.4	39.68	23.18	70.50
**0.7**	**0.5**	**39.87**	**23.32**	**70.75**

## Data Availability

The original data presented in the study are openly available in the Refer-ASV dataset at https://pan.quark.cn/s/6923ca46d245 (accessed on 8 February 2026). The released data include all real-world ASV navigation videos and their corresponding tracking annotations, while referring expression annotations are currently provided for a subset of the dataset. The complete dataset, including all referring expressions, will be released at the same link upon completion of the open-source review process of our laboratory.

## Abbreviations

The following abbreviations are used in this manuscript:

ASV	Autonomous Surface Vehicle
RMOT	Referring Multi-Object Tracking
MOT	Multi-Object Tracking
TBD	Track-by-Detection
HOTA	Higher-Order Tracking Accuracy
MOTA	Multiple Object Tracking Accuracy
IDF1	IDentity F1 Score
CNN	Convolutional Neural Network
LLM	Large Language Model
MLP	Multilayer Perceptron
FPS	Frames Per Second
FLOPs	Floating-Point Operations
GPU	Graphics Processing Unit
CPU	Central Processing Unit
API	Application Programming Interface
CUDA	Compute Unified Device Architecture
OMBS	One-More-Boat Sample Strategy
SAFM	Scale-Semantic-Aware Fusion Module
VOQM	Value-Aware Object Query Management
